# Diagnostic testing for pulmonary tuberculosis in low and middle-income countries: innovations to achieve universal access—a narrative review

**DOI:** 10.1016/j.eclinm.2026.104232

**Published:** 2026-09-22

**Authors:** Nazir A. Ismail, Rebecca H. Berhanu, Nina von Knorring, Marguerite Massinga Loembe, Mikashmi Kohli, Rumina Hasan, Siva K. Shanmugam, Surbhi Sheokand, Achilles Katamba, Madhukar Pai, Shaheed V. Omar

**Affiliations:** a Department of Clinical Microbiology and Infectious Diseases, National Health Laboratory Service and Faculty of Health Sciences, University of Witwatersrand, Johannesburg, South Africa; b Vanderbilt University Medical Center, Division of Infectious Diseases, Department of Medicine, Nashville, TN, USA; c Marguerite Massinga Loembe, Consultant Heath Systems Strengthening and Diagnostics, Matahari Global Solutions, Malaysia; d Foundation for Innovative New Diagnostics, Geneva, Switzerland; e Aga Khan University, Pakistan; f London School of Tropical Medicine and Hygiene, London, United Kingdom; g ICMR-National Institute for Research in Tuberculosis, Chennai, India; h McGill International TB Center and McGill School of Population and Global Health, McGill University, Montreal, Canada; i Clinical Epidemiology & Biostatistics Unit, Department of Medicine, Makerere University College of Health Sciences, Kampala, Uganda; j Centre for Tuberculosis (National & WHO Supranational TB Reference Laboratory), National Institute for Communicable Diseases, A Division of the National Health Laboratory Services, Johannesburg, South Africa

**Keywords:** Tuberculosis, Diagnostics, Access, LMIC, Near point of care

## Abstract

Though rapid molecular diagnostics for tuberculosis (TB) have substantially improved diagnostic accuracy and turnaround times, implementation and impact has been constrained by cost, infrastructure requirements, and continued reliance on sputum. This review examines advances in diagnostic technologies and sampling strategies that could overcome these barriers and accelerate universal access to TB diagnostics. We conducted a narrative review of studies published between Jan 1, 2020, and April 30, 2026, focusing on near-point-of-care molecular (NPOC) platforms, next-generation lipoarabinomannan (LAM) assays, and non-sputum sampling approaches. Emerging NPOC molecular platforms, demonstrate high sensitivity using both sputum and tongue-swab specimens while substantially reducing equipment and testing costs compared with existing nucleic acid amplification test platforms. Next-generation urine LAM assays offer improved sensitivity and potential for use beyond people living with HIV. In addition, parallel testing approaches with multiple sample types, including sputum, tongue swab, stool for children, and urine testing, can substantially increase diagnostic yield in specific populations. Integrating low-cost technologies with non-sputum sampling strategies could provide a scalable pathway towards universal access to TB diagnostics. Realizing this potential will require streamlined regulatory pathways, sustainable financing, and integration of these tools into primary healthcare systems.

**Funding:**

None.

## Introduction

Early care-seeking, accurate and timely diagnosis, and prompt treatment initiation are essential to interrupt TB transmission and reduce TB-related mortality. However, diagnostic delays remain a major barrier to achieving the End TB Strategy targets, particularly in high-burden, low- and middle-income countries (LMICs). Such delays are consistently associated with increased risk of severe disease, ongoing transmission, and higher mortality,^1^, ^2^, ^3^ and possible increased risk of post-TB lung disease.^4^^,^^5^

Patient-related delays vary widely by settings, with median total delays from symptom onset to diagnosis of 28 days reported in LMICs, compared with 10 days in high TB burden upper-middle-income countries.^6^ There is strong evidence across studies that female sex and greater distance to health facilities are strongly associated with longer patient delays.^6^ Additional contributing patient factors include mild or insidious onset of TB symptoms leading to delayed recognition of illness, limited TB awareness, lower educational attainment, transportation costs, out-of-pocket payments for testing, care-seeking from multiple primary care providers (e.g., pharmacists, private providers, or traditional and informal practitioners), perceived stigma, and financial insecurity.^6^, ^7^, ^8^, ^9^, ^10^ In many settings, initial care-seeking outside the national TB program—particularly in private or traditional sectors—has been associated with substantial diagnostic delays.^6^ For women, delays often stem from de-prioritizing their health amid caregiving demands, power imbalances, and limited financial autonomy. For men, delays more often reflect financial pressures, work constraints, and stigma that discourage timely care-seeking.^6^^,^^11^, ^12^, ^13^

Health system-related delays are also longer in LMICs than in upper middle income countries (14 vs 4 days).^6^ Contributing factors include limited decentralization of diagnostics—particularly in lower-level or rural facilities—the lack of affordable, easy-to-use point-of-care TB tests, resource constraints, stigma, low clinical suspicion for TB, and laboratory delays.^6^ Low clinical suspicion is particularly critical: fewer than half of patients presenting with TB symptoms are offered testing, highlighting inadequate symptom screening and failure to test symptomatic individuals as key drivers of delay.^7^ These gaps reflect frontline providers’ limited awareness of national programme expectations or their inability to adhere to case-finding guidelines amid heavy workloads and competing clinical priorities.^7^ Standardized patient studies across multiple countries further show that primary care providers frequently prescribe empirical antibiotics rather than pursue TB testing in symptomatic patients.^14^ Diagnostic delays are particularly consequential in extrapulmonary TB, which is often missed, especially at the primary care level, leading to delayed diagnosis and complications.^15^, ^16^, ^17^, ^18^ Criticially, lack of access to molecular diagnostics with resistance detection capacity contributes to empiric initiation of first-line anti-TB treatment, risking amplification of drug resistance in individuals with undetected resistance.^19^

In this review, we examine the importance and impact of expanding access to near-patient diagnostics, and highlight three emerging developments to improve access: i) new technologies suitable for use at the primary health care (PHC) level, ii) alternative sample types to overcome the challenge of sputum scarcity iii) reduced test costs and improved country processes to support early adoption of new diagnostics.

## Search strategy and selection criteria

This is a narrative review of diagnostic assays for the diagnosis of pulmonary tuberculosis (TB) disease. The review focuses on a) technologies relevant to decentralized near-point-of-care (NPOC) and point-of-care (POC) use; and b) alternative (non-sputum) sample types. Outcomes of interest include diagnostic accuracy (sensitivity and specificity), diagnostic yield, and where available, implementation and cost-effectiveness data. We include technologies that are commercially available or in late-stage development with clear pathways to implementation. We do not include tests for TB infection (e.g., latent TB or incipient TB), screening-only algorithms or imaging-based diagnostics. Populations of interest include adults, children, people living with HIV (PLHIV), and individuals with sputum scarcity, reflecting groups in whom alternative sampling strategies and decentralized testing are particularly relevant.

A narrative review methodology was used to summarize current evidence in the field. We conducted a PubMed search for publications between 01 January 2020 and 30 April 2026, limited to English-language articles and including relevant preprints. Primary search terms included “TB,” “tuberculosis,” and “pulmonary tuberculosis,” supplemented with section-specific terms such as “diagnosis,” “testing,” “detection,” “non-sputum,” “near point of care,” “point of care,” “rapid testing,” “sensitivity,” “specificity,” “performance,” “primary health care,” and “community,” as well as terms relating to patient pathways and diagnostic delays (e.g., “symptoms,” “delayed diagnosis,” “diagnostic delay,” “impact,” and “outcome”).

Targeted searches were also performed for specific specimen types (e.g., urine, saliva), diagnostic technologies (e.g., Xpert, Truenat, PlusLife) and test mechanisms (e.g., lipoarabinomannan, nucleic acid amplification test). Titles and abstracts were independently screened by two reviewers using Covidence (NI, RB), with eligible studies undergoing full-text review prior to inclusion. Where recent high-quality systematic reviews were available, these were used as primary sources and supplemented with subsequently published primary studies.

Additional evidence sources included World Health Organization (WHO) guidance documents, dashboards, and other global policy resources, as well as the diagnostics database maintained by the Foundation for Innovative New Diagnostics (FIND).

## Access to rapid molecular diagnostics and impact on diagnostic delays and outcomes

The introduction of WHO-recommended rapid diagnostics (WRDs) represents a major advance in efforts to reduce TB diagnostic delays and improve case detection. In 2010, the WHO recommended Xpert MTB/RIF (Cepheid, United States) as the initial diagnostic test for TB, replacing smear microscopy due to its improved sensitivity and ability to detect rifampicin resistance. This recommendation was expanded in 2020–2021 to include newer molecular assays, such as Xpert MTB/RIF Ultra and Truenat MTB & RIF Dx (Molbio, India).^20^

Expanded access to WRDs has improved both individual and population-level outcomes. Meta-analyses show that WRDs reduce diagnostic delays by approximately 2 days and half the time to diagnosis compared to smear microscopy.^21^^,^^22^ Evidence for reduced mortality is less consistent: Xpert MTB/RIF has not demonstrated a significant reduction in all-cause mortality overall (RR 0.89, 95% CI 0.75–1.05), athough mortality benefits are likely among people living with HIV (PLHIV) (OR 0.80, 95% CI 0.67–0.96).^23^^,^^24^ At a population-level, however, longitudinal studies consistently associate expansion expansion of rapid molecular testing with reductions in TB mortality, likely reflecting the cumulative benefits of earlier diagnosis, reduced transmission, and more timely treatment initiation.^25^ Economic evaluations support government investment in scale-up. In South Africa, national investment in molecular TB dignoatics generated an estimated US $6.4 billion (95% uncertaintly interval 4.8–8.0) in societal benefits from an investment of US$315 million.^26^ Together, these findings suggest that the full clinical, epidemiological, and economic benefits of WRDs depend on widespread, decentralized access and effective integration into TB care pathways.

While molecular testing improves individual and population outcomes, there are important efficiency trade-offs relating to scale-up and decentralization. In the XPEL-TB cluster-randomized trial in Uganda, placing Xpert instruments at peripheral health facilities increased same-day diagnosis and timely treatment initiation compared with referral-based testing but reduced efficiency at low-volume sites.^27^ Similarly, the TB-CAPT trial in Mozambique and Tanzania found that decentralized Truenat testing reduced diagnostic turnaround time, increased same-day treatment initiation, and lower patient out-of-pocket costs compared with centralized Xpert testing.^28^^,^^29^ However, incremental programmatic costs were high ($1332 per additional treatment initiation; range $763–$5558), reflecting lower instrument utilization and higher capital costs associated with greater decentralization.^29^

Despite compelling evidence for the benefits of WRDs, access remains a major barrier to realizing their full impact. In 2024, only 54% of people newly diagnosed TB were tested using a WRD.^20^ Coverage remains particularly limited in high-burden settings, with only 8 of 30 high TB burden countries reporting WRD availability at more than half of TB diagnostic sites.^20^ To accelerate scale-up, WHO introduced the Universal Access to Rapid TB Diagnostics Standard and the accompanying TB Benchmark Dashboard to monitor national progress.^30^ However, achieving universal access will require diagnostic platforms that are not only accurate but also affordable, deployable at lower levels of the health system, and scalable in diverse settings.

## Improving primary care facility level access through new near-patient iTechnologies

WHO classifies TB molecular diagnostics according to operational complexity (low, moderate, and high). Although low-complexity automated nucleic acid amplification tests (LC-aNAATs), including Xpert and Truenat, have expanded access to rapid molecular diagnosis, their deployment ramins constrained by related to infrastructure requirements, environmental operating conditions (requirements for heat and humidity control), maintenance needs, and most importalty cost.^31^ As these platforms have been extensively reviewed elsewhere, they are not discussed further.

A new generation of point-of-care (POC) and near-point-of-care (NPOC) assays has emerged that address many of these barriers, although none of the currently available platforms detect drug resistance. The WHO target product profiles define POC tests as simple diagnostic assays that can be performed at or near the site of patient care with minimal infrastructure and provide results during the same clinical encounter.^32^ NPOC systems are molecular platforms that are compact, portable, battery-operable, simpler to use and less expensive than conventional LC-aNAATs.^33^

Development of POC and NPOC diagnostics is expanding rapidly. The FIND pipeline directory lists 73 technologies for TB diagnostic confirmation comprising 51 NPOC and 22 POC platforms, yet relatively few have been evaluated in peer-reviewed studies.^34^ The following sections review the available evidence for these emerging NPOC molecular and POC non-sputum diagnostic platforms.

### New near point of care molecular tests for TB

Unlike current WHO-recommended LC-aNAAT platforms which rely on real-time PCR, emerging NPOC molecular tests use isothermal amplification, a simpler, and lower-cost approach that is well-suited to decentralized, resource-limited settings. The operational characteristics, sample requirements, complexity, and drug resistance detection capabilities of the reviewed NPOC platforms are summarized in Table S1 (Supplementary Appendix).

The most extensively evaluated NPOC platform is MiniDock (PlusLife, China), a swab-based assay that can be performed using sputum or tongue swab specimens. WHO recommended MiniDock in early 2026.^33^ MiniDock demonstrated high diagnostic accuracy with both specimen types, although sputum generally outperformed tongue swabs (sputum sensitivity 85%, tongue swab sensitivity 76%, Table 1).^33^^,^^40^ Sensitivity appears lower among people living with HIV (79%) and individuals with low mycobacterial burden,^55^ and evidence for its performance in asymptomatic individuals and community settings remains limited.^39^

**Table 1:** Diagnostic performance of TB diagnostic tests by specimen type.

Specimen	Technology	Population	Sensitivity (95% CI)	Specificity (95% CI)	Ref
**NAAT platforms**					
LC-aNAAT platforms—Sputum					
Sputum	Xpert Ultra	Presumtive TB	91% (88–93)	95% (93–96)	35
Sputum	Xpert Ultra	PLHIV	88% (82–92)	95% (92–97)	35
Sputum	Xpert Ultra	HIV-negative	90% (85–94)	96% (91–98)	35
Sputum	Xpert Ultra	Children (sputum), age 1–4 years	75% (63–83)	95% (92–97)	36
Sputum	Xpert Ultra	Children (sputum), age 5–9 years	82% (65–92)	95% (92–98)	36
Sputum	Truenat MTB Plus	Presumptive TB	91% (84–95)	96% (95–97)	37
Sputum	Truenat Ultima	Presumptive TB	90% (85–93)	85% (80–88)	38
Sputum	Truenat Ultima	Presumptive TB/ACF	84% (76–89)	97% (96–88)	39
Near-point-of-care NAAT—Sputum					
Sputum	MiniDock	Presumptive TB	85% (82–88)	98% (97–98)	33,40
Sputum	MiniDock	PLHIV	79% (69–87)	97% (95–98)	33,40
Sputum	EasyNAT MTC	Presumptive TB	93% (88–99)	95% (90–100)	41
LC-aNAAT platforms—Tongue swab					
Tongue swab	Xpert Ultra	Presumptive TB	66% (61–70)	99% (98–99)	42
Tongue swab	Truenat Ultima	Presumptive TB/ACF	74% (64–82)	98% (96–99)	39
Near-point-of-care NAAT—Tongue swab					
Tongue swab	MiniDock	Presumptive TB	76% (71–81)	99% (99–100)	33,40
Tongue swab	MiniDock	PLHIV	69% (58–79)	99% (98–100)	33,40
LC-aNAAT platforms—Urine					
Urine	Xpert Ultra	PLHIV	33% (27–39)	98% (97–99)	43
LC-aNAAT platforms—Stool					
Stool	Xpert Ultra	Children <15 years (vs MRS)	73% (53–81)	–	44
Stool	Xpert Ultra	Children <15 years (vs TB clinical case definition)	38% (22–56)	–	44
Stool	Xpert Ultra	PLHIV adults	24% (16–32)	94% (92–96)	45
Stool	Xpert Ultra	PLHIV adults, CD4 ≤200 cells/μL	46% (24–68)	–	45
Stool	Xpert Ultra	PLHIV adults, CD4 >200 cells/μL	22% (13–32)	–	45
**LF-LAM antigen tests**					
First generation LF-LAM					
Urine	Determine TB-LAM	PLHIV with presumptive TB	42% (31–55)	91% (85–95)	46,47
Urine	Determine TB-LAM	PLHIV unselected	35% (22–50)	95% (89–96)	39,41
Urine	Determine TB-LAM	PLHIV hospitalized, symptomatic	52% (40–64)	87% (78–93)	39,41
Urine	Determine TB-LAM	HIV-negative	11% (6–18)	92% (89–95)	48
Urine	Determine TB-LAM	Children with HIV (<15 years)	47% (33–60)	77% (57–96)	49
Urine	Determine TB-LAM	Children without HIV (<15 years)	32% (8–57)	79% (63–96)	50
Next-generation LF-LAM					
Urine	SILVAMP TB LAM II	PLHIV	56% (33–76)	95% (92–96)	51,52
Urine	BP-LAM	Symptomatic	63% (58–69)	93% (90–96)	53,b
Urine	BP-LAM	PLHIV	67% (58–76)	90% (83–94)	53,b
Urine	BP-LAM	HIV-negative	62% (55–69)	96% (92–99)	53,b
Sputum	PATHFAST TB LAM	Case control study	75% (59–87)	a95% (75–100)	54

Comparator data for sputum Xpert Ultra and urine Determine TB-LAM provided for reference. All percentages rounded to nearest whole number.

Sensitivity and specificity reported relative to microbiological reference standard (MRS) unless otherwise noted.

MRS, microbiological reference.

a PATHFAST TB LAM test was positive in 26/40 NTM-culture positive samples.

b Preprint.

The EasyNAT MTC assay (Ustar Biotechnologies, China) demonstrated comparable sensitivity to Xpert MTB/RIF in a single-site study in China.^41^ A more portable, lower-cost version (PortNAT) is in development. Multiple additional NPOC NAAT systems are in the pipeline, including FlashDetect (Coyote, China), SARAGENE (CoSara, India), and UniAmp (Huwel Lifesciences, India), though published clinical evidence for these is not yet available.^34^

### New point of care lipoaribinomman assays

Lipoarabinomannan (LAM) assays are lateral flow (LF) immunoassays that detect a *Mycobacterium tuberculosis* cell wall antigen. They simple, “urine dipstick”-like tests are readily deployable in primary care settings but have substantially lower sensitivity than sputum-based NAATs. Determine TB-LAM remains the only commercially available LF-LAM assay and has limited sensitivity outside advanced HIV disease (45% in PLHIV and 56% in individuals with CD4 counts ≤100 cells/μL; Table 1).^46^^,^^47^ Its clinical benefit is greatest among hospitalized PLHIV with advanced immunosuppression, where it has been shown to reduce mortality.^56^ An additional limitation is cross-reactivity with non-tuberculous mycobacteria (NTM), particularly disseminated NTM disease in advanced HIV.^57^ Although next generation LAM assays use alternative antibody targets that my reduce cross-reactivity, evidence remains limited.

Next generation POC LAM assays aim to improve sensitivity by incorporating pre-analytic urine concentration and signal amplification technologies.^58^ The Silvamp TB LAM assay (Fujifilm, Japan) was the first next-generation platform to approach commercialization. Although initial lot-to-lot variability delayed implementation, the updated version (Silvamp TB LAM II) provides an alternative to Determine TB-LAM among PLHIV, but has not demonstrated substantially improved performance (Table 1, Table S1).^51^^,^^52^ Clinical data on cross-reactivity with NTM are not yet available.

BP-LAM (Biopromic AB, Sweden) incorporates urine concentration and novel monoclonal antibodies to improve LAM detection. BP-LAM has higher sensitivity than Determine TB-LAM and consistent performance across HIV status and CD4 count strata, supporting its potential for broader use however, current evidence remains limited to preprint data and requires further validation (63% sensitivity overall, Table 1).^53^ Specificity was also improved compared with Determine TB-LAM (93% vs 89%), although early data suggest possible cross-reactivity in patients with disseminated NTM infection.

PATHFAST TB LAM (PHC Corporation, Japan) is the first sputum-based LAM assay and has primarily been evaluated as a treatment-monitoring tool rather than a standalone diagnostic test. Early studies demonstrate promising sensitivity, although cross-reactivity with NTM is a concern.^54^ Additional LF-LAM assays in late stage development for which published clinical performance data is not yet available include Standard F TB LAM (SD Biosensor, Korea), and Fluxus LAM (Fluxus, United States).^34^

## Expdning access through patient-friendly sample types and parallel testing strategies

Beyond advances in diagnostic technologies, improving access to TB testing also depends on using sample types that are easier to obtain.^59^ Although sputum remains the cornerstone of pulmonary TB diagnosis, many individuals—including the very ill, PLHIV, children, and asymptomatic individuals—are unable to produce an adequate specimen.^60^ Diagnostic yield, defined as the proportion of people in whom TB is identified among all people eligible for testing, is therefore an important metric alongside diagnostic accuracy.^61^ Alternative sample types, used alone or in parallel with sputum, can increase diagnostic yield by capturing cases that would otherwise be missed. The WHO now recommends urine, stool and tongue swab testing in selected populations. This section focuses on the use of non-sputum specimens across existing diagnostic platforms (not limited to NPoC technologies) and emphasizes the additional diagnostic yield achieved through paralell testing strategies.

### Urine

Urine offers a practical advantage over sputum in terms of sample accessibility. A systematic review among adult PLHIV (≥15 years) found that 98% could provide urine compared with 82% for sputum, increasing to 99% vs 54% among unselected individuals tested irrespsective of TB symptoms.^59^ Although urine Determine TB-LAM had lower overall diagnostic yield than sputum Xpert Ultra (41% vs 61%), yields were comparable among unselected hospitalized PLHIV (51% vs 47%), and paralell testing increased overall case detection to 71%.^59^ Similarly a Cochrane review demonstrated that the addition of urine LF-LAM to sputum Xpert increased diagnostic yield from 71% to 78% with only a modest reduction in specificity.^47^ On this basis, WHO now strongly recommends concurrent urine LF-LAM and sputum NAAT testing for adults with HIV, with a conditional recommendation for children with HIV due to concerns about loss of specificity.^47^^,^^62^

Urine Xpert Ultra has also shown promise. A seven-country study among PLHIV found sensitivity comparable to Determine TB-LAM, but higher specificity (Table 1).^43^ Unlike LF-LAM assays, urine Xpert Ultra provides rifampicin susceptibility results; although at greater cost and operational complexity.

### Stool

Diagnosing TB in children is challenging, and gold-standard gastric aspiration is invasive and requires skilled healthcare workers. Stool NAAT has emerged as a practical alternative, with simplified centrifuge-free processing methods, including the single-step (SOS) protocol, improving feasibility at primary health care level.^63^ As a standalone test, stool Xpert Ultra has only moderate sensitivity (Table 1, 73% vs microbiological references standard, 38% vs clinical TB case definition).^44^ However, its greatest value lies in combination testing. Testing two stool samples rather than one increased performance by 20–25%,^64^ while combining stool with respiratory-specimen NAAT substantially improves case detection compared with either specimen alone. A Cochrane review found that parallel stool and respiratory testing achieved 80% sensitivity.^62^ Similarly, a landmark Thai study found that combinations of minimally invasive specimens (two nasopharyngeal aspirates or one nasopharyngeal aspirate plus one stool sample) matched the diagnostic yield of two gastric aspirates, while adding urine further improved case detection.^65^ On this basis, the WHO now recommends concurrent respiratory and stool NAAT testing for all children with presumptive TB, including those living with HIV.^66^

Evidence for stool testing in adults remains more limited; performance is influenced by HIV status and degree of immunosuppression, with highest sensitivity in individuals with advanced HIV (Table 1, 46% in PLHIV with CD4 < 200 cells per μL vs 21% in those with higher CD4).

### Oral samples

Oral sampling offers a non-invasive alternative for individuals unable to produce sputum and where sputum induction is not feasible. Detailed performance data for MiniDock and Truenat Ultima on tongue and saliva swabs are presented in Table 1; broader evidence is summarized here. Performance varies considerably by anatomic site and population. Tongue swabs consistently outperforming cheek swabs and oral rinses.^67^ Additionally, sensitivity is lower in asymptomatic individuals and in children (Table 1).^68^, ^69^, ^70^ Among sputum scarce individauls, tongue swabs identified TB in 18–25% of cases, supporting a complementary role when sputum cannot be obtained.^70^ Morning collection appears to improve sensitivity, and methodological refinements—including high-capacity foam swabs, centrifugation, and magnetic bead DNA concentration—have further improved performance relative to sputum reference standards.^71^^,^^72^ However, most studies have evaluated tongue swabs in symptomatic, sputum-producing adults. Evidence in the populations most likely to benefit—including sputum-scarce individuals, children, and community screening populations—remains limited.

## Improving access to affordable technologies and the accelerating pathways to country introduction

### Cost-effective decentralization requires optimized testing strategies

Cost-effectiveness of TB diagnostics is not determined by test price alone but is highly sensitive to testing volume, instrument placement, and linkage to care. Economic modeling studies demonstrate that cost-effectiveness drops significantly in low-volume settings, where the cost per TB diagnosis can exceed seven times that of high-volume centers, highlighting critical volume thresholds for instrument placement decisions.^73^^,^^74^ Triage strategies that combine low-cost screening tools with confirmatory molecular testing offer an alternative model: digital chest X-ray with computer-aided detection (dCXR/CAD) followed by confirmatory Xpert can reduce screening costs by approximately half compared with universal molecular testing, with only modest reductions in diagnostic yield.^75^ For symptomatic individuals, combining CRP-based screening with confirmatory Xpert has also been shown to be more cost-effective than universal molecular testing.^76^ Taken together, these findings suggest that the path to cost-effective decentralization lies not in universal molecular testing alone, but in tiered, algorithm-based strategies that match test complexity and cost to the clinical and programmatic context. Integrating TB testing with HIV, respiratory virus and STI diagnostics on shared paltforms could further improve cost-.^77^

### Affordable technologies are changing the economics of decentralization

Cost remains a major barrier to TB diagnostic access in LMICs. Although the negotiated ex-works price of Xpert MTB/RIF Ultra was reduced to ∼US$10 per test, it remained unchanged for almost a decade. The first competing molecular platform, Truenat, lowered the per-test cost only marginally to approximately US$8, still above the target price of <US$5.^32^ Instrument and warranty costs remain a major constraint to decentralization, ranging from approximately US$17,000–90,000 for Xpert and US$11,000–18,000 for Truenat, particularly in low-volume settings.^78^ Modeling suggests that achieving a cost-effectiveness threshold of <US$500 per disability-adjusted life year (DALY) averted will require substantial reductions in instrument and warranty costs.^78^

Emerging NPOC platforms have the potential to fundamentally change these economic tradeoffs. PlusLife test costs are US$3.60, and instrument costs are a fraction of those of current technologies (∼US$350 vs ∼ US$10,000).^79^ These price points could make decentralized molecular testing cost-effective even in low-volume primary healthcare settings, though trade-offs related to drug-resistance detection and the need for reflex testing must be carefully considered.

### A changing innovation landscape

After decades of reliance on donor funding, many high-TB-burden countries are confronting shrinking international aid budgets. At the same time, innovation from the Global South is expanding access to affordable technologies. Lower-cost molecular platforms, tongue-swab sampling, and AI-assisted screening tools are now approaching large-scale implementation. Analysis of WHO-endorsed diagnostics and products listed through WHO Prequalification, the Global Fund Expert Review Panel for Diagnostics (ERPD), or the Global Drug Facility shows an increasingly diverse market, with technologies originating from China, India, Japan, and South Korea. Furthermore, FIND's diagnostic pipeline indicates that almost two-thirds (64%; 48/75) of NPOC and POC technologies are expected to originate from Africa, Southeast Asia, and the Western Pacific.^34^ Harnessing this growing regional innovation capacity will be critical to closing the diagnostic gap and accelerating progress towards ending TB.

### Accelerating country introduction

Beyond affordability, regulatory, policy and health system barriers continue to delay implementation. Critical Pathway Analyses conducted across nine high-TB-burden countries in Africa and Asia identified prolonged delays in national policy adoption and continued dependence on imported diagnostics, with reported delays of 2–8 years in parts of Africa.^80^^,^^81^ In many countries, limited regulatory capacity for diagnostics means that national approvals depend heavily on WHO recommendations. India provides an important exception, with a regulatory framework that has enabled rapid approval and deployment of locally developed technologies such as Truenat and the Pathodetect MTB Rif Inh assay (Mylab, India).

Regulatory systems are gradually strengthening. Five of the nine countries assessed now have well-functioning regulatory systems for vaccines and medicines, while the Africa Medicines Agency is advancing regional regulatory harmonization. The successful pilot cross-continental listing process for MPOX diagnostics demonstrates a model that could be applied to TB. Global mechanisms—including the ERPD, Global Fund early-access initiatives, and regional pooled procurement platforms—may accelerate access, reduce costs, strengthen supply chains, and ultimately support local manufacturing.

Realizing the potential of decentralized NPOC testing will also require sustained investment in the health system infrastructure needed for scale-up, including laboratory networks, connectivity and electronic result reporting. Full integration with national TB programmes will also be crucial to enable surveillance, real-time monitoring of diagnostic performance, improve supply chain management, and strengthen linkage to care.^82^^,^^83^ As donor funding declines, increased domestic investment in both diagnostic technologies and the systems required to implement them will be essential to overcome chronic underinvestment in TB programmes and fully realize the potential of NPOC technologies.^84^ This momentum was underscored at the Global Implementation Workshop on NPOC TB Diagnostics in Bangkok (8–10 July 2026), co-hosted by the Stop TB Partnership and the Global Fund, where representatives from 13 countries outlined plans collectively targeting 3 million NPOC tests, marking an important step towards country-level introduction.^85^

## Outstanding questions and future directions

Although emerging low-cost NPOC technologies, coupled with easier-to-collect sample types, offer scalable and decentralized solutions, important gaps remain. The real-world and population-level impact of these technologies on access, reducing time to treatment and clinical outcomes requires further evaluation. Evidence for NPOC use in children remains limited, particularly for alternative sample types such as tongue swabs, which require further optimization and validation. Tongue swabs also offer potential value for screening, but their performance in low-bacterial-load samples in such scenarios warrants additional study.

Beyond current modalities, greater investments are needed to advance true POC testing. The growing pipeline of urine-based POC diagnostics is encouraging and should be rapidly evaluated in clinical settings. There is also a great need for investment in blood-based testing to address the practical limitations of urine- and stool-based approaches. Another key access issue is taking testing out of health providers' hands and into patients' hands, allowing them to self-test. However, realizing the potential of patient self-testing will require continued maturation of oral- and urine-based assays to minimize biohazardous waste handling. Recent innovations offer strong potential for improving equitable access to high-quality diagnostics, and doubling down on these efforts to achieve a POC self-test should be the next milestone.

## Contributors

NAI, RHB, MP and SVO conceptualized the paper. NAI and RHB led the literature search and drafted the initial manuscript, with section contributions from ML, MP, SVO, MK and NVK. All authors reviewed the search terms and the manuscript design, provided critical revisions, contributed intellectual content and approved the final version for publication. NAI had responsibility for the decision to submit the manuscript for publication.

## Data sharing statement

No new data were generated. All data analyzed in this review are derived from previously published studies, which are cited within the article.

## Declaration of interests

NAI institution received a grant to evaluate the performance, feasibility and costs of implementing the MiniDock MTB Tests (PlusLife, China) in public sector laboratories in South Africa. MP serves as an advisor to these non-profit organizations: the Gates Foundation, the World Health Organization, and Partners in Health. ML received consultancy fees to perform the critical pathway analysis in 5 African countries. All other authors declare no conflicts of interest.
