# Supplementary material for: Diagnostic testing for pulmonary tuberculosis in low and middle-income countries: innovations to achieve universal access—a narrative review
Source: eClinicalMedicine. 2026 Sep 22;100:104232. doi: 10.1016/j.eclinm.2026.104232 (PMC13659590; doi:10.1016/j.eclinm.2026.104232)
Supplement: Supplementary file 1 [file mmc1.docx]

**Table S1: Comparison of workflow, portability, turnaround time, and drug-resistance detection across selected emerging TB diagnostic technologies.**

| **Instrument** | **Technology** | **Sample preparation (steps/time)** | **Operator skill required** | **Portability** | **Result turnaround time** | **Drug resistance detection** |
| --- | --- | --- | --- | --- | --- | --- |
| **MiniDock PlusLife MTB** | NPOC - Isothermal NAAT. (RNase hybridization–assisted amplification) | Simple sample preparation. 2 steps: (1) Swirl swab in releasing agent; (2) Automated bead-beating lysis + thermal lysis via Thermolyse device. ~2-3 min hands-on. | **Low** | **High**  Small, battery operable, portable device (<1kg) | **15-30 min** | **No** –  MTB detection only; no rifampicin resistance |
| **EasNAT MTC** | NPOC - Isothermal NAAT (cross-priming amplification) | Manual sample preparation steps: sputum processing with kit reagents, then add to reaction cartridge. No centrifuge needed; ~10–15 min hands-on. | **Moderate**  Basic laboratory skills | **High**  Compact bench-top system, battery operable | **45–60 min** | **No** -  MTB detection only; no rifampicin resistance |
| **Stool Xpert MTB/RIF Ultra** | LC-NAAT – Real-time PCR | Multiple manual preparation steps (stool processing + reagent incubation); ~15–20 min hands-on. | **Moderate** | **Low** (Xpert instrument required) | **~90 min** | **Yes** rifampicin resistance detection |
| **Urine Xpert MTB/RIF Ultra** | LC-NAAT – Real-time PCR | Centrifugation/concentration required, followed by Xpert sample preparation; relatively labour-intensive | **Moderate–High** | **Low**  Xpert instrument required | **~90 min** | **Yes** rifampicin resistance detection |
| **SILVAMP FujiLAM (v2)** | Lateral flow immunoassay - silver-amplified monoclonal Ab detection of LAM | Multiple manual steps with timed reagent additions with 40 min waiting time in between steps, then add to cartridge. Visual lateral-flow interpretation. | **Moderate** | **Very high** | **~60 min** | **No** |
| **PathFast TB-LAM** | Automated chemiluminescent enzyme immunoassay quantitative LAM | Minimal urine preparation; automated cartridge assay. Digital/fluorescence readout provides objective positive/negative result. | **Low** | **Moderate** Portable analyser required | **~15–20 min** | **No** |
| **BP-LAM** | POC urine LAM | No preparatory steps. Lateral flow dipstick assay with short waiting time (~20-30 minutes). Visual lateral-flow interpretation. Final commercial implementation and interpretation procedures have not yet been fully described. | **Low** | **Very high** | **20–30 min** | **No** |
